# Overcrowded housing during adolescence and future risk of premature mortality: a 28-year follow-up of 556,191 adolescents from Switzerland

**DOI:** 10.1016/j.lanepe.2023.100667

**Published:** 2023-06-15

**Authors:** Sarah M. Mah, Laura C. Rosella, Mika Kivimäki, Cristian Carmeli, the SNC study group

**Affiliations:** a Dalla Lana School of Public Health, University of Toronto, Toronto, Canada; b UCL Brain Sciences, University College London, London, UK; c Clinicum, University of Helsinki, Helsinki, Finland; d Population Health Laboratory (#PopHealthLab), University of Fribourg, Fribourg, Switzerland

**Keywords:** Crowded housing, Premature mortality, Social determinants of health, Causal inference, Adolescence and young adulthood

## Abstract

**Background:**

Few large-scale studies have examined the health impacts of overcrowded housing in European countries. The aim of this study was to assess whether household crowding during adolescence increases the risk of all-cause and cause-specific mortality in Switzerland.

**Methods:**

Study participants were 556,191 adolescents aged 10–19 years at the 1990 census from the Swiss National Cohort. Household crowding at baseline was measured as the ratio between the number of persons living in the household and the number of available rooms, categorized as none (ratio ≤ 1), moderate (1 < ratio ≤ 1.5), and severe (ratio > 1.5). Participants were linked to administrative mortality records through 2018 and followed for premature mortality from all causes, cardiometabolic disease and self-harm or substance use. Cumulative risk differences between ages 10 and 45 were standardized by parental occupation, residential area, permit status and household type.

**Findings:**

Of the sample, 19% lived in moderately and 5% lived in severely crowded households. During an average follow-up of 23 years, 9766 participants died. Cumulative risk of death from all causes was 2359 (95% compatibility intervals: 2296–2415) per 100,000 persons when living in non-crowded households. Living in moderately crowded households led to 99 additional deaths (−63 to 256) per 100,000 persons and living in severely crowded households 258 additional deaths (−37 to 607) per 100,000 persons. The effect of crowding on mortality from cardiometabolic diseases, self-harm or substance use was negligible.

**Interpretation:**

Excess risk of premature mortality in adolescents living in overcrowded households appears to be small or negligible in Switzerland.

**Funding:**

University of Fribourg Scholarship Programme for foreign post-doctoral researchers.

**Research in context**

**Evidence before this study**

We performed a systematic search on the Medical Literature and Retrieval System Online (MEDLINE) database for studies investigating the association or causal relationship between household overcrowding during childhood or adolescence and premature mortality in adulthood published from inception to February 2023. The search strategy and the eligibility criteria for study selection are presented in Supplementary Table S1. The search included MeSH terms and free text related to overcrowded housing, social or socioeconomic determinants of health, childhood or adolescence, and mortality.

Studies were included if conducted in countries, regions or cities that are part of the European Economic Area (EEA) and the Organization for Economic Co-operation and Development (OECD). Only studies in English were included. A total of 213 articles were screened based on titles and abstracts, 32 based on full text, and eight were included in this systematic review. Studies were conducted in Finland, Norway and the United Kingdom. Definitions for the period of childhood and adolescence varied. Finnish studies examined household crowding when respondents were 0–14 years old, while 3 Norwegian studies examined overcrowding up to 24 years old. Time of baseline data ranged from 1937 to 1975, and follow-up spanned 1948 to as late as 2010. All studies examined associations between household overcrowding and all-cause mortality, five included specific causes of death, and one study focused on all-cause death after myocardial infarction. Definitions of crowding also varied substantially in the number of people per room (ranged from <1 to 2 people per room considered *not crowded*) as well as the types of rooms considered in the measure (e.g. heated rooms, rooms in regular use). Overall, the evidence suggests a modest relationship between higher household overcrowding in childhood and adolescence and higher risk of death in adulthood. However, some studies were limited to a few individual-level covariates and relied on unadjusted or minimally adjusted models. In half the studies, household crowding was included in a composite housing measure, rather than assessed independently for its association with mortality.

**Added value of this study**

Beyond studies conducted in Finland, Norway, and the UK assessing household overcrowding during early-life encompassing childhood and adolescence prior to 1975, there is no evidence of its association with premature mortality when overcrowding occurs specifically during adolescence in more recent years and in other European countries. This nation-wide study of 556,191 contemporary Swiss adolescents followed from 1990 (10–19 years old at baseline) until 2018 (38–47 years old) leveraged population-based census and administrative data that permitted a focus on household crowding in adolescence, adjustment for a range of individual-level sociodemographic factors, and ascertainment of all-cause premature mortality in addition to specific causes of death hypothesized as being most relevant to early-life household overcrowding. Additionally, we adopted a causal framework involving transparent identification of our assumptions as well as analytic approaches aimed at mitigating bias. While the findings of this study demonstrate small differences in mortality across different levels of household crowding, there was little evidence to suggest that overcrowding in adolescence impacts premature deaths from all causes, from self-harm and substance use, or cardiometabolic disease for contemporary Swiss adolescents.

**Implications of all the available evidence**

Our findings, together with previous reports, indicate that the contribution of household overcrowding in adolescence toward premature mortality is minimal or negligible for European countries such as Switzerland. Research efforts should be directed towards other adverse early-life exposures, and their relationship to mortality and other measures of health and well-being across the life course.

## Introduction

Childhood and adolescence are periods of rapid growth and development that are considered critical or sensitive to health later in life.^1^ Studies have linked environmental constraints and other adverse circumstances in childhood and adolescence to worse educational outcomes,^2^ less favourable cardiovascular, metabolic, and inflammatory biomarkers,^3^^,^^4^ and increased premature mortality.^5^^,^^6^ Environmental constraints during adolescence could be a significant limiting factor during which individuals are sensitive to social cues and developing a sense of independence as well as emotional and social cognitive capacity.^7^^,^^8^ One feature of adverse circumstances, highlighted by the WHO and OECD, is household overcrowding, although the importance of this constraint as a risk factor for health in high-income European countries remains unclear.

Household overcrowding occurs when a dwelling space is too small to accommodate the number of people in a household and is considered an important socioeconomic indicator of housing insecurity and material deprivation.^9^ Overcrowding is a risk factor for psychological distress,^10^ poor self-rated health,^11^ poor mental health,^12^, ^13^, ^14^, ^15^, ^16^ as well as communicable and non-communicable diseases.^17^^,^^18^ In historical registry studies from Finland and the UK, overcrowding has been linked to increased risk of myocardial infarction^19^ and mortality.^20^^,^^21^ Although household standards have increased in Europe, interest in household overcrowding has persisted both as an independent measure of socioeconomic disadvantage and as a component in many composite indices of deprivation.^22^ Such indicators have been employed in studies of mortality for a number of countries, including Switzerland.^23^

The prevalence of overcrowding in Swiss cities and agglomerations ranged from 3.8% in Lucerne to 18.5% in Geneva in 2020.^24^ A Swiss study showed an association between household overcrowding and lower educational attainment in adolescents aged 15–18 years and young adults aged 20–24 years.^14^ However, little is known about the impacts of household overcrowding in adolescence on long-term health and mortality outcomes in Switzerland. Premature death is an important international health indicator and has been recognized as a feasible and cross-cutting target for reduction by the United Nations Sustainable Development Goals.^25^ After accidental death, suicide (self-harm) is the most common cause of death for Swiss adolescents,^26^ and could be affected by adverse social conditions, including household overcrowding. Unstable housing has been associated with drug-related mortality in the general population,^27^ while a longitudinal study demonstrated a link between childhood socioeconomic status with substance use and substance use-related deaths.^28^ Early life adversity may also contribute to the development of metabolic disorders and cardiovascular disease.^29^ The aim of this study was to assess the effect of household overcrowding in adolescence on future risk of premature mortality due to all-causes, self-harm and substance use, and cardiometabolic disease.

## Methods

### Data source

The Swiss National Cohort (SNC) is a population-based longitudinal study constructed using baseline sociodemographic information from the 1990 census. Study participants are linked to electronic records of continually updated national censuses, migration and mortality files.^30^ Record linkage for the SNC was performed using a combination of deterministic and probabilistic approaches.^31^^,^^32^ The 1990 Swiss census contains information on individual sociodemographic characteristics, household characteristics (e.g. number of people living in the household, number of rooms, children, housing tenure) and building characteristics (e.g. dwelling type, age of building, number of floors), and has an estimated coverage rate of 98.6%. Mortality data were derived from death certificates of the mortality register in Switzerland, which contains date of death, immediate cause of death, underlying cause of death and secondary causes related to concomitant diseases. Follow-up was available through the 31st of December 2018.

### Target, study and analytic populations

**Fig. 1:**
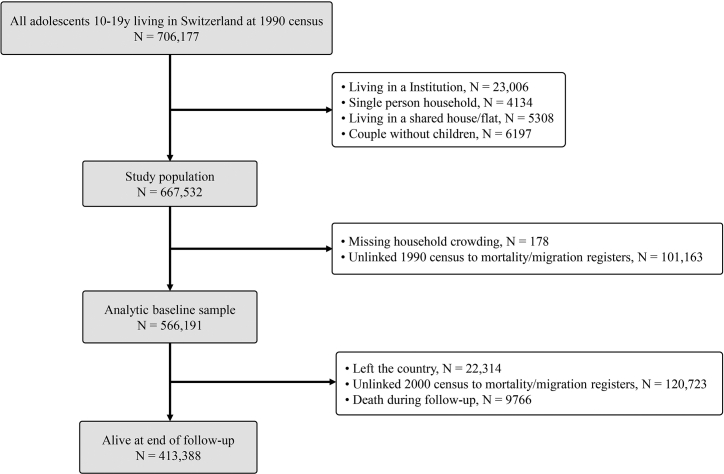
**Flow chart of the participant selection into the study and follow-up of the analytic sample.** Participants who could not be reliable linked to mortality registers after 2000 census were right censored after 10 years of follow-up.

The target population was adolescents living with their parents in Switzerland. The study population was adolescent respondents of the SNC aged 10–19 years (i.e., born between 1971 and 1980) and living with their parents at the time of 1990 census (N = 667,532, Supplementary Figure S1). The analytic sample was derived by excluding individuals for whom the number of available rooms was unknown (N = 178) and those for whom records from the 1990 census could not be reliably linked to mortality registers (N = 101,163) due to the probabilistic nature of the record linkage.^30^ Thus, the analytic cohort included 556,191 participants (see Fig. 1 and Supplementary Figure S1).

### Causal model, exposure, outcome, and covariates

**Fig. 2:**
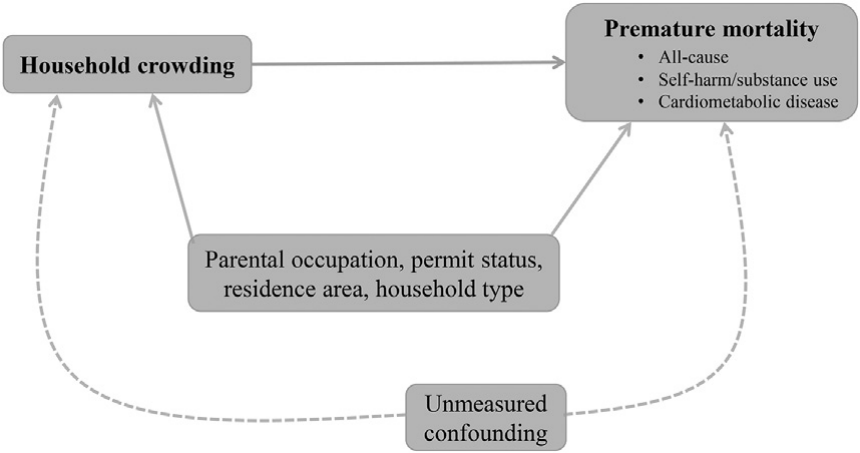
**Causal model**. Household crowding is the exposure and premature mortality related to all causes, self-harm/substance use, and cardiometabolic disease are the outcomes. Measured confounders include living in a 1- or 2-parent household, parental occupation (low/high position), permit status (Swiss vs non-Swiss), and urban/non-urban area of residence. Unmeasured confounding is with dashed arrows. For the sake of simplicity, censoring due to loss at follow-up is not drawn.

Our causal model in Fig. 2 focuses on premature mortality related to all causes, self-harm/substance use and cardiometabolic disease (outcomes) as driven by living in overcrowded housing during adolescence (exposure). Using information in the household and dwelling section of the 1990 census, we defined household crowding as living in a household with more than one person per room, measured as the ratio between the number of persons living in the household and the number of available rooms excluding kitchen and bathrooms (for the distribution of this ratio, see Supplementary Figure S2). We then produced a 3-level measure of household crowding and assigned values below 1 as households with no crowding, values between 1 and 1.5 as those with moderate crowding, and values >1.5 as those with severe crowding.^33^ In sensitivity analyses, we operationalized crowding (No/Yes) based on an alternative definition from Eurostat.^34^

According to our causal model, we identified four measured potential confounders which we included in the analysis. These variables were parental occupational position (“low” when both parents were unemployed or had unskilled professions, “high” otherwise), residential area type (urban, non-urban– which includes both peri-urban and rural areas), permit status (Swiss when at least one of the parents declared to be a Swiss citizen, non-Swiss otherwise), and household type (single-parent, two-parent). In addition to these four measured confounders, we identified three unmeasured potential confounders (parental wealth, parental income, offspring disability) as part of the minimally sufficient adjustment set in a detailed causal model (Supplementary Figure S3). The relationship between adverse childhood exposures and health may vary across different sociodemographic^35^ and geographic contexts.^36^ Therefore, we also examined these covariates as potential effect modifiers of the relationship between household crowding and premature mortality by stratifying analyses across levels of the four measured covariates.

Our outcomes were premature mortality (defined as death before the age of 75 years)^37^ from all causes, mental health related causes (self-harm and substance use encompassing alcohol and drugs), and cardiometabolic diseases (cardiovascular disease and endocrine, nutritional, and metabolic diseases). Follow-up was until 31 December 2018 when the participants reached age 38–47 years for those alive and not lost at baseline or during follow-up. Specific causes of death were identified using the *International Classification of Diseases (ICD), 8*th *Revision* (deaths occurring before 1995, 14% of total decedents) and *10*th *Revision.* Detailed ICD codes corresponding to the examined causes of death are reported in Supplementary Table S2.

### Statistical analysis

The effect of moderate or severe household crowding was estimated using marginal risk differences and ratios between age 10 and 45 with respect to no household crowding. The cumulative risk of dying due to household crowding was computed as the predicted proportion of decedents based on the counterfactual scenario of every study participant living in a non-, moderately, or severely crowded household without loss during follow-up. Risk of premature death was estimated via complement of Kaplan–Meier survival (for all-cause mortality) or Aalen-Johansen cumulative incidence function (for cause-specific mortality) with age as the timescale. We standardized these risks by measured confounding via stabilized inverse probability weights (IPWs).^38^ Weight diagnostics encompassing the quality of the weights and the balance of measured confounders across exposure levels were examined (see Supplementary Material). The standardized mean difference for these confounders was <0.01 after IPW, indicating the sample was well-balanced across levels of household crowding. Potential misspecification of the IPW model was ascertained in sensitivity analyses by estimating models using incrementally truncated weights. Internal validity of effect estimates relies on a set of assumptions: consistency; no interference; positivity; no residual confounding; no measurement error of the exposure, outcome, or confounders; and correct specification of the statistical estimation model^39^ (for a detailed description of these assumptions, see Supplementary Material). For external validity, we considered IPWs for individuals lost at baseline and follow-up in a sensitivity analysis (see Fig. 1 and Supplementary Material, Tables 3 and 4).

Compatibility intervals were generated via percentiles of 500 block bootstrap draws with replacement. A block corresponded to a household (N = 407,429). Within each bootstrapped sample, the effect estimates were the average of 30 multiply imputed data sets (nearly 5% missing parental occupational position and 0.2% missing permit status) using multiple imputation by chained equations (see Supplementary Material). Finally, ICD-10 codes that are considered vague, imminent or immediate causes of death, or cannot be underlying causes of death (often collectively termed “garbage codes”, 14% of all deaths, see Supplementary Table S5) were re-assigned to either deaths related to mental health, cardiometabolic diseases or any other cause of death via a data-driven predictive model based on elastic net regression^40^ (for a detailed description of this approach see Supplementary Material).

All analyses were conducted using R 4.0.2 software.

### Role of the funding source

The funder had no role in the study design, data collection, analysis and interpretation, report writing, or decision to submit for publication.

## Results

### Characteristics of the analytic sample

Baseline characteristics of the sample by household crowding and overall are reported in Table 1. Participants had a median age of 14.4 years and 52% were male. A small proportion of the participants lived in severely crowded households (5%), while 19% lived in households considered moderately crowded. Most participants lived in two-parent households (90%), had parents of high occupational position (80%), were of Swiss nationality (79%) and lived in non-urban areas (75%). A greater proportion of individuals who lived in severely crowded households during adolescence were from families with two parents (96% compared with 90% overall), had parents who held “lower” occupational positions (42% compared with 15% overall), had non-Swiss permit status (64% compared with 21% overall), and lived in urban areas (35% compared with 25% overall). Over a mean follow-up time of approximately 23.4 years, 9766 deaths from all causes, 711 deaths (7% of all decedents) from cardiometabolic diseases, and 3222 deaths (33% of all decedents) from suicide or substance use were identified.

**Table 1:** Characteristics of the analytic sample at baseline and follow-up for all and stratified by household overcrowding.

	Total N = 566,191	Household Crowding		
		No N = 427,965 (76%)	Moderate N = 108,604 (19%)	Severe N = 29,622 (5%)
**Age** [years]	14.4 (12.1 to 16.8)	14.5 (12.2 to 16.9)	14.2 (11.9 to 16.4)	14.2 (11.9 to 16.5)
**Sex**				
Female	271,531 (48%)	205,105 (48%)	52,077 (48%)	14,349 (48%)
Male	294,660 (52%)	222,860 (52%)	56,527 (52%)	15,273 (52%)
**Household type**				
2 parents	507,574 (90%)	374,176 (87%)	105,093 (97%)	28,305 (96%)
1 parent	58,617 (10%)	53,789 (13%)	3511 (3%)	1317 (4%)
**Parental occupation**				
High	454,139 (80%)	365,655 (85%)	73,678 (68%)	14,806 (50%)
Low	85,221 (15%)	43,328 (10%)	29,482 (27%)	12,411 (42%)
Missing	26,831 (5%)	18,982 (4%)	5444 (5%)	2405 (8%)
**Parental permit of residence**				
Swiss	446,568 (79%)	370,665 (87%)	65,545 (60%)	10,358 (35%)
Non-Swiss	118,697 (21%)	56,892 (13%)	42,777 (39%)	19,028 (64%)
Missing	926 (0.2%)	408 (0.1%)	282 (0.3%)	236 (0.8%)
**Residence area**				
Urban	140,982 (25%)	98,957 (23%)	31,599 (29%)	10,426 (35%)
Non-Urban	425,209 (75%)	329,008 (77%)	77,005 (71%)	19,196 (65%)
**Mortality**				
All-cause	9766	7510 (77%)	1760 (18%)	496 (5%)
Self-harm/substance use	3222 (33%)	2552 (79%)	537 (17%)	133 (4%)
Cardiometabolic disease	711 (7%)	545 (77%)	123 (17%)	43 (6%)
Other causes	5833 (60%)	4413 (76%)	1100 (19%)	320 (5%)
Mean follow-up time, years (SD)	23.4 (8)	23.3 (8)	23.7 (8)	23.7 (8.2)
Follow-up person-years [100,000]	132.4	99.7	25.7	7
Crude mortality rate, deaths per 100,000 PY	73.7	75.3	68.4	70.6

Age distribution is described as median (1st to 3rd quartile).

Across levels of household crowding, standardized mean difference for household type, parental occupation, parental permit of residence and residence area at 1990 census was 0.24, 0.56, 0.79 and 0.18, respectively.

### Household overcrowding and premature mortality

**Fig. 3:**
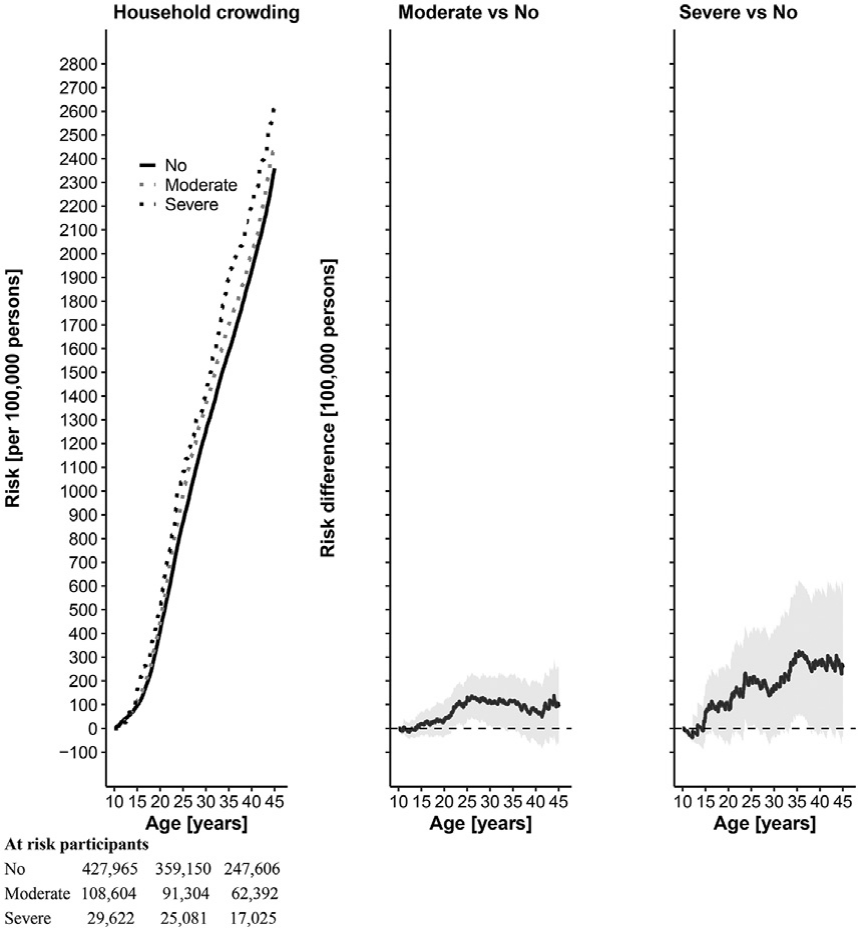
**Cumulative risk of mortality from all causes and effect of household crowding.** All-cause mortality cumulative incidence or risk per 100,000 persons between age 10 and 45 is reported for no, moderate, and severe household crowding. Risks were standardized by living in a 1- or 2-parent household, parental occupation, parental permit status (Swiss vs non-Swiss), and urban/non-urban area of residence. The number of at risk participants for each level of household crowding is reported at age 10, 25 and 40 at the bottom of the first column. Cumulative risk differences per 100,000 persons for moderate vs no crowding and severe vs no crowding are reported in the second and third column, respectively.

With respect to all-cause premature mortality, risk differences by level of household crowding suggest small effects of living in overcrowded households (Table 2 and Fig. 3). While living in non-crowded households led to a cumulative risk of 2359 (95% compatibility intervals: 2296 to 2415) deaths from all causes per 100,000 persons, living in moderately crowded households led to 99 additional deaths per 100,000 persons (95% CI: −63 to 256). Living in severely overcrowded households rendered 258 additional deaths per 100,000 persons (95% CI: −37 to 607). This corresponded to risk ratios of 1.04 (0.97 to 1.11) and 1.11 (0.99 to 1.26), respectively. Compared to living in a non-crowded household, living in a severely crowded household appeared to lead to higher additional deaths when having parents with low occupational position (Δ = 289 (−358 to 919) per 100,000 persons), living in an urban area (Δ = 362 (−328 to 1089) per 100,000 persons), and having single parent (Δ = 842 (−666 to 2381) per 100,000 persons), compared to those having parents with high occupational position, living in a non-urban area, or having two parents, respectively (Supplementary Table S6). However, these estimates were imprecisely estimated and thus inconclusive.

**Table 2:** Estimated effect of household crowding on all-cause and cause-specific premature mortality.

	All causes	Cardiometabolic	Suicide/Substance Use
No crowding	2359 (2296 to 2415)	210 (191 to 231)	760 (730 to 789)
Moderate vs no crowding	RD: 99 (−63 to 256) RR: 1.04 (0.97 to 1.11)	RD: −31 (−74 to 16) RR: 0.85 (0.66 to 1.08)	RD: −4 (−83 to 77) RR: 0.99 (0.89 to 1.10)
Severe vs no crowding	RD: 258 (−37 to 607) RR: 1.11 (0.99 to 1.26)	RD: −8 (−92 to 89) RR: 0.96 (0.56 to 1.45)	RD: 14 (−162 to 197) RR: 1.02 (0.79 to 1.25)

Risk in the reference category (no crowding), risk differences per 100,000 persons (RD) and risk ratios (RR) by deaths related to all causes, cardiometabolic diseases and suicide/substance use (95% compatibility intervals).

Risk between age 10 and 45 is standardized by parental occupational position, permit status, household type, and area of residence.

Finally, the effect of household crowding on cardiometabolic or suicide/substance use related deaths was negligible (Table 2).

### Sensitivity analyses

Results were largely unchanged in models utilizing an alternative definition of household crowding based on Eurostat (Supplementary Table S7). When incrementally truncating the weights, findings were in line with those from the main analysis (Supplementary Table S8), although effect estimates were slightly smaller. This suggests that the effect sizes reported in the main analysis may slightly overestimate the true effect of household crowding because of potential IPW model misspecification. Estimates were in line with those from the main analyses when we included IPWs for individuals lost at baseline and follow-up (Supplementary Table S9), suggesting that selection bias due to those losses was negligible.

## Discussion

This nation-wide study leveraged a large population-based cohort with 28 years of follow-up to examine the extent to which household overcrowding may impact premature mortality in Switzerland. Although we observed some small differences in mortality across different levels of household crowding, the overall evidence suggested that overcrowding in adolescence would have little impact on all-cause mortality, or deaths from self-harm and substance use, or cardiometabolic disease in this population.

This study was motivated by previous work demonstrating associations between household overcrowding and a broad array of health-related outcomes,^10^, ^11^, ^12^, ^13^, ^14^, ^15^, ^16^, ^17^, ^18^, ^19^ many of which were related to psychosocial distress, mental health as well as chronic diseases. Our results contrast findings from Finland,^20^ Norway,^41^ and the UK^21^ to an extent–as some of these studies present unadjusted or minimally adjusted estimates and/or use different thresholds and reference categories for overcrowding. However, our results agree with other literature.^14^^,^^19^^,^^42^ For example, a Finnish study demonstrated a persistent relationship between household crowding in childhood and a 16–25% increased risk of myocardial infarction,^19^ but did not establish a conclusive relationship to short- or long-term mortality after myocardial infarction. With respect to mental health related outcomes, a previous Canadian study found no association between household crowding during childhood or adolescence and psychological distress,^42^ noting that overcrowding might be too unstable an exposure in adolescence for an association to have been found. In a New Zealand cohort, the presence of a cross-sectional, but lack of a longitudinal association with psychological distress suggested that household crowding may induce stress and mental health at the time of overcrowding, but these negative impacts did not persist into later life.^14^

The strengths of this study include its large sample size of over half a million individuals and an extended follow-up of 28 years for an objectively assessed outcome derived from a national mortality register. Our exposure variable was assessed using two standard definitions of household crowding that have been widely utilized and validated, and correlates with other measures of socioeconomic status.^9^ Our application of a causal inference framework to estimate marginal risk differences and overcome known biases related to traditional hazard ratio estimates^43^^,^^44^ is a novel and robust approach to studies of household crowding and health.

This study also bears limitations. Our study is open to selection bias due to individuals lost at baseline and during follow-up. Although these losses may have slightly reduced the precision of effect estimates, the results from a sensitivity analysis suggest bias, if any, may be negligible. Our findings may not be transportable to adolescents in living arrangements such as group homes or shared houses. Adolescents who do not live with their parents may comprise a more deprived and vulnerable segment of the population which could compound the effect of household crowding on premature mortality for this group, as has been found for lower income groups in previous studies.^10^ As pointed out previously,^42^ our exposure variable is open to some misclassification, because a single baseline measure does not capture residential moves to more or less crowded households during adolescence. Moreover, our measure does not capture all aspects of household crowding, such as the physical area available to occupants, or whether and with whom adolescents share space with (i.e., siblings, parents or others). To reduce possible misclassification of the outcomes, we used previously described approaches and re-assigned certain non-informative or unspecific codes to the death causes under examination.^40^^,^^45^

Lastly, our observational study is subject to residual confounding by unmeasured or imprecisely measured confounders, as we were limited by the bias-variance trade-off and information collected by census and administrative data. For example, parental wealth was not available although it is likely to be an important socioeconomic determinant of housing and health outcomes, including premature mortality.^46^ Furthermore, our data did not include information on children's disabilities, which may contribute to confounding bias via an unblocked backdoor path with parental income.

In conclusion, this study shows evidence for a small or negligible effect of household crowding in adolescence on premature mortality in Switzerland. The relationship between housing and health is complex and multifaceted,^47^ and overcrowding may be an important characteristic of housing in terms of health and quality of life that is nevertheless limited in scale in relation to premature mortality. Overall, this study provides an important message to direct research attention towards other potentially important early-life exposures, and suggests a potential need to examine a broader range of relevant outcomes related to health and psychosocial well-being.^48^

## Contributors

CC and SM conceptualised and designed the study. CC and SM contributed to the statistical design of the study and CC conducted the data analyses. CC and SM had direct access to the dataset. SM and CC interpreted the data. SM and CC conducted the literature search and SM wrote the first draft of the manuscript. All authors critically revised and approved the final version of the manuscript. CC had final responsibility for the decision to submit for publication.

## Data sharing statement

Individual data from different data sets were used for the construction of the SNC. All these data are the property of the SFSO and can only be made available by legal agreements with the SFSO. This also applies to derivatives such as the analysis files used for this study. However, after approval of the SNC Scientific Board, a specific SNC module contract with the SFSO would allow researchers to receive analysis files for replication of the analysis.

## Declaration of interests

The authors declare no conflicting interests.
