# Supplementary material for: Overcrowded housing during adolescence and future risk of premature mortality: a 28-year follow-up of 556,191 adolescents from Switzerland
Source: Lancet Reg Health Eur. 2023 Jun 15;31:100667. doi: 10.1016/j.lanepe.2023.100667 (PMC10300403; doi:10.1016/j.lanepe.2023.100667)
Supplement: Supplementary Figures S1–S3 and Tables S1–S9 [file mmc1.pdf]

SUPPLEMENTARY MATERIAL: Overcrowded housing during adolescence and future risk of premature mortality: a 28-year follow-up of 556 191 adolescents from Switzerland

Sarah M. Mah[1], Laura Rosella[1], Mika Kivimäki[2,3], Cristian Carmeli[4], for the SNC study group

[1] Dalla Lana School of Public Health, University of Toronto, Toronto, Canada

[2] UCL Brain Sciences, University College London, London, UK

[3] Clinicum, University of Helsinki, Helsinki, Finland

[4] Population Health Laboratory (#PopHealthLab), University of Fribourg, Fribourg, Switzerland

Research in Context 3
Supplementary Table 1. 3
References of included studies 4
Methods 5
ICD-10 garbage codes re-allocation approach 5
Assumptions for internal validity of effect estimates 6
IPW models 8
Imputation model 8
STROBE Checklist for Cohort Studies 9
Supplementary Figure 1 11
Supplementary Figure 2 12
Supplementary Figure 3 13
Results 14
Supplementary Table 2 14
Supplementary Table 3 15
Supplementary Table 4 16
Supplementary Table 5 17
Supplementary Table 6 18
Supplementary Table 7 19
Supplementary Table 8 19
Supplementary Table 9 20
Supplementary References 20

RESEARCH IN CONTEXT

Supplementary Table 1.

Search strategy and eligibility criteria for systematic search.

Database: Medical Literature Analysis and Retrieval System Online (MEDLINE; PubMed interface)

**SEARCH STRATEGY**		
(Crowding[Mesh] OR crowd* OR overcrowd* OR housing)<br>AND<br>(Adverse Childhood Experiences*[Mesh] OR Social Determinants of Health*[Mesh] OR Socioeconomic Factors*[Mesh])<br>AND<br>(adult*)<br>AND<br>(child* OR adolescen*)<br>AND<br>("Mortality, Premature"[Mesh] OR "Cause of Death"[Mesh] OR Mortality[Mesh])		
**ELIGIBILITY CRITERIA**		
	**INCLUSION**	**EXCLUSION**
**STUDY DESIGNS**	Observational cohort.<br>Systematic reviews and meta-analyses<br>Quasi-experiment<br>Experimental randomized and non-randomized controlled trials (parallel/cluster/cross-over)	Case-control, and cross-sectional studies, case reports, case series<br>Opinion papers, letters to the editor, comments, conference proceedings, policy papers, study protocols, and animal studies<br>Pre-post comparisons without control groups, or other uncontrolled studies will be excluded.
**PARTICIPANTS**	Individuals exposed in childhood or adolescence (≤19 years)	Otherwise.
**EXPOSURE**	Observational study: household crowding<br>Quasi-experiment / experiment: intervention affecting household crowding	Otherwise.
**COMPARATOR**	Observational study<br>Household crowding levels, or<br>Levels of housing status = composite measure including crowding	Otherwise.
**OUTCOME MEASURES**	Mortality overall or cause-specific (any cause) in adulthood	Otherwise.
**TIME FRAME**	No restriction by duration of follow-up.	Not applicable.
**SETTING**	Studies conducted in the European Economic Area (EEA) and the Organisation for Economic Co-operation and Development (OECD) (see list below).	Otherwise.

	Both regional-wide (e.g. one city or one county only) and country-wide studies will be included.<br>List of countries: Australia, Austria, Belgium, Bulgaria, Canada, Chile, Colombia, Costa Rica, Croatia, Cyprus, Czechia, Denmark, Estonia, Finland, France, Germany, Greece, Hungary, Iceland, Ireland, Israel, Italy, Japan, Latvia, Liechtenstein, Lithuania, Luxembourg, Malta, Mexico, Netherlands, New Zealand, Norway, Poland, Portugal, Romania, Slovakia, Slovenia, South Korea, Spain, Sweden, Switzerland, Turkey, United Kingdom, United States.	
**LANGUAGE**	English	Otherwise.

References of included studies

1. Claussen B, Davey Smith G, Thelle D. Impact of childhood and adulthood socioeconomic position on cause specific mortality: the Oslo Mortality Study. Journal of Epidemiology and Community Health. 2003;57(1):40.
2. Dedman DJ, Gunnell D, Smith GD, Frankel S. Childhood housing conditions and later mortality in the Boyd Orr cohort. Journal of Epidemiology and Community Health. 2001;55(1):10-5.
3. Kilpi F, Silventoinen K, Konttinen H, Martikainen P. Early-life and adult socioeconomic determinants of myocardial infarction incidence and fatality. Social science & medicine. 2017;177:100-9.
4. Kuh D, Hardy R, Langenberg C, Richards M, Wadsworth MEJ. Mortality in adults aged 26-54 years related to socioeconomic conditions in childhood and adulthood: post war birth cohort study. BMJ. 2002;325(7372):1076.
5. Martikainen P, Elo I, Tarkiainen L, Mikkonen J, Myrskylä M, Moustgaard H. The changing contribution of childhood social characteristics to mortality: a comparison of Finnish cohorts born in 1936–50 and 1961–75. International Journal of Epidemiology. 2020;49(3):896-907.
6. Myrskylä M, Elo IT, Kohler IV, Martikainen P. The association between advanced maternal and paternal ages and increased adult mortality is explained by early parental loss. Social science & medicine. 2014;119:215-23.
7. Næss Ø, Claussen B, Davey Smith G. Relative impact of childhood and adulthood socioeconomic conditions on cause specific mortality in men. Journal of Epidemiology and Community Health. 2004;58(7):597.
8. Næss Ø, Claussen B, Smith GD. Housing conditions in childhood and cause-specific adult mortality: The effect of sanitary conditions and economic deprivation on 55,761 men in Oslo. Scandinavian journal of public health. 2007;35(6):570-6.

METHODS

ICD-10 garbage codes re-allocation approach

Mortality data were retrieved from death certificates in Switzerland, which contain the date of death, the immediate cause of death, the underlying cause of death (UCOD), and concomitant diseases. The immediate cause of death is defined as "the terminal event" before death and the UCOD as the "disease or injury that initiated the events resulting in death". Death certificates are filled by a certifying physician at the time of death. Causes of death and concomitant diseases are reviewed and coded by the Swiss Federal Statistical Office (SFSO) according to International Classification of Diseases (ICD) standards.

In our sample of 706 177 adolescents living in Switzerland at 1990 census (See Figure 1) 10 500 deaths occurred, of which 81% of ICD codes could be mapped to an UCOD while 19% of deaths corresponded to ICD-10 codes labeled as garbage codes (1). Garbage codes occur when physicians do not comply with international recommendations in the completion death certificates. Garbage codes are codes that are either too vague for public health relevance (e.g. unspecified cancer), are either imminent or immediate causes of death, (e.g. septicemia), or are codes that cannot be plausible causes of death (e.g. back pain). Garbage codes are problematic because they bias mortality statistics due to potential misclassification of death causes, therefore they misinform health care policy making. Garbage codes are classified into four levels related to the potential severity of public health policy implications resulting from their misclassification from level 1 (very high implication) to level 4 (low implication).

We therefore reassigned garbage codes to valid UCODs. Level 3 and 4 garbage codes (N = 494, 4·7% of ICD-10 coded deaths) were re-allocated manually to specific death categories following ICD-10 chapters. Level 1 and 2 garbage codes (N = 1504, 14·3% of ICD-10 coded deaths) were reassigned using multinomial elastic net regression (2). The regression model included the following predictors: age of death, sex, household type, living environment at 1990 census (urban, peri-urban, rural), linguistic region at 1990 census (German, French, other), country of birth, year of birth, month of birth, season of birth, immediate and intermediate cause of death, and concomitant diseases. The outcome variable corresponded to three death categories related to: i) cardiovascular and diabetes, urogenital, blood, and endocrine diseases, ii) related to substance use disorders and self-harm, iii) all other deaths (see Supplementary Table 2).

First, we developed a classification model using the part of the dataset containing valid UCODs. This dataset was randomly split into a training set and a test set of equal size. On the training set, we fit 11 models with optimal lambdas identified through 10-fold cross-validation for alpha values ranging from 0 to 1 by 0·1 increment. An alpha value of 0 is a pure ridge regression, while an alpha value of 1 is a pure lasso regression. On the test set, we predicted outcomes by applying our previously generated 11 models, and we chose the model with the lowest misclassification rate of approximately 8%. Second, we predicted UCODs using the previously built model on the part of the dataset containing garbage codes. Death categories before and after garbage codes reallocation are reported in Supplementary Table 5.

Assumptions for internal validity of effect estimates

Internal validity of effect estimates based on the potential outcomes framework relies on a set of assumptions: consistency; no interference; positivity; no residual confounding; no measurement error of the exposure, outcome, or confounders; and correct specification of the statistical estimation model.

The consistency assumption requires a clear and specific definition of the exposure and that the difference between the exposure as measured and the exposure as intervened upon is minimal (3). In our case, household overcrowding is specific (i.e. does not include various socioeconomic dimensions as typically done for defining neighborhood socioeconomic indices). However, overcrowding could be intervened upon with different policies. For example, housing policies to promote housing affordability might produce different effects on premature mortality than policies promoting construction of more spacious and/or affordable accommodations. Our causal estimates might be interpreted as an average of the effects of these different 'types' of interventions.

The 'no interference' assumption postulates that a given intervention received by members of one household does not affect the mortality risk of individuals living in other households. In order to account for the potential 'spillover' effects of the housing conditions/interventions of one household to members of different households (e.g., a beneficial housing intervention could have indirect effects on non-household family members, social contacts), one might use multi-level regression models to accommodate this assumption (4). However, multi-level models can only be reliably estimated when, on average, five or more members per household are available (5). In our study, the average number of adolescents per household was lower, 1·33, making this statistical

approach unfeasible. However, the potential bias ensuing from a violation to this assumption in some localized households might be negligible in our study, as we estimated effects from all Swiss households sampled in the 1990 census.

We aimed to address the (stochastic) positivity assumption – which states that there must be both exposed and unexposed individuals at every level of the measured confounding variables – by limiting the number of categories within included variables where possible, by stabilizing our IPWs and by evaluating the absence of extreme values in the estimates of the weights. We ensured that the values of the weights were smaller than 10.

Like all observational studies, our study is subject to residual confounding as a result of unblocked backdoor paths (see Supplementary Figure 3) and because we limited the number of categories of the measured confounders. However, we anticipate that two unmeasured confounders (parental wealth and child disabilities) that are positively associated with both overcrowding and mortality would likely strengthen the effect estimates of crowding on premature mortality provided in our study. Therefore, our findings are likely to be conservative.

Measurement error might be present due to the probabilistic linkage, due to misclassification of exposure and confounders as they were self-reported or measured only once during adolescence, and due to misclassification of the cause-specific mortality because of the garbage ICD-10 codes. However, the bias due to misclassification related to probabilistic linkage is likely small (6). Misclassification of exposure and confounders due to self-report is also likely small or negligible as they were reported by the parents themselves, not by their offspring later in life as in most retrospective studies of mortality at older ages. Finally, we aimed to mitigate misclassification of the examined mortality causes by re-allocating garbage codes as described in previous section. The re-allocation model provided reasonably low misclassification rates of the outcome (<10%).

We addressed the assumption of correct specification of the estimator model by running a sensitivity analysis related to the IPW model, which is described in the next section. Since the Kaplan-Meier or Aalen-Johansson estimators are non-parametric, there is no need to ascertain their specification. The sensitivity analysis indicated that the effect sizes reported in the main analysis may slightly overestimate the true effect of household crowding because of potential IPW model misspecification.

IPW models

Stabilized inverse probability weights (IPWs) were obtained using the entropy balancing method (7), whereby predictors of the exposure included household type, parental occupational position, residential area and parental residence permit. We specified these predictors as linearly additive - that is, we did not include interaction terms. We implemented the models via the R package WeightIt (8). We ran a sensitivity analysis to assess potential IPW model misspecification by incrementally truncating the weights, as suggested by Cole and Hernan (9). Weights had means of nearly one and a maximum value smaller than six. Weights obtained via the entropy balancing method displayed higher effective sample size and smaller variability than weights obtained using multinomial logistic regression. The standardized mean difference for all selected confounders (i.e. the examined exposure predictors) was smaller than 0·01 after weighting.

In a sensitivity analysis to assess potential selection bias due to individuals lost at baseline or during follow-up, weights were the product of IPWs for loss at baseline, measured confounding and loss during follow-up weights. The IPW model for loss at baseline included parental education, occupational position, permit status, residence area and household crowding at 1990 census (Supplementary Table 3), because for these covariates a standardized mean difference larger than 0·1 was observed between the proportion of the sample lost at baseline and the analytic sample. The IPW model for follow-up losses included the parental education and residence area at 1990 census (see Supplementary Table 4) and was specified as a Cox proportional hazard model via the ipw R package.

Imputation model

Within each bootstrapped sample, the effect estimates were the average of 30 imputed data sets whereby, under the hypothesis of missingness at random, chained equations were used to impute missing data in parental occupational position, parental education and permit status (see Table 1). The imputation model included the following predictors: residential area type at 1990 census, household crowding and type at 1990 census, language area (German, French or other) of the household at 1990 census, sibling status (single child or not), age at 1990 census, and the cumulative hazard. Imputations were performed with the mice R package.

STROBE Checklist for Cohort Studies

	Item No	Recommendation	Page No
**Title and abstract**	1	(*a*) Indicate the study's design with a commonly used term in the title or the abstract	1,2
		(*b*) Provide in the abstract an informative and balanced summary of what was done and what was found	2
**Introduction**			
Background/rationale	2	Explain the scientific background and rationale for the investigation being reported	5,6
Objectives	3	State specific objectives, including any prespecified hypotheses	6
**Methods**			
Study design	4	Present key elements of study design early in the paper	6,7
Setting	5	Describe the setting, locations, and relevant dates, including periods of recruitment, exposure, follow-up, and data collection	6-8
Participants	6	(*a*) Give the eligibility criteria, and the sources and methods of selection of participants. Describe methods of follow-up	6,7
		(*b*) For matched studies, give matching criteria and number of exposed and unexposed	
Variables	7	Clearly define all outcomes, exposures, predictors, potential confounders, and effect modifiers. Give diagnostic criteria, if applicable	6-8
Data sources/ measurement	8	For each variable of interest, give sources of data and details of methods of assessment (measurement). Describe comparability of assessment methods if there is more than one group	6-8
Bias	9	Describe any efforts to address potential sources of bias	8-9
Study size	10	Explain how the study size was arrived at	6,7
Quantitative variables	11	Explain how quantitative variables were handled in the analyses. If applicable, describe which groupings were chosen and why	6,7
Statistical methods	12	(*a*) Describe all statistical methods, including those used to control for confounding	8,9
		(*b*) Describe any methods used to examine subgroups and interactions	8
		(*c*) Explain how missing data were addressed	8,9
		(*d*) If applicable, explain how loss to follow-up was addressed	8
		(*e*) Describe any sensitivity analyses	7,8
**Results**			
Participants	13	(a) Report numbers of individuals at each stage of study—eg numbers potentially eligible, examined for eligibility, confirmed eligible, included in the study, completing follow-up, and analysed	6,7,14
		(b) Give reasons for non-participation at each stage	6,7,14
		(c) Consider use of a flow diagram	14
Descriptive data	14	(a) Give characteristics of study participants (eg demographic, clinical, social) and information on exposures and potential confounders	9, 17
		(b) Indicate number of participants with missing data for each variable of interest	8
		(c) Summarise follow-up time (eg, average and total amount)	9,17
Outcome data	15	Report numbers of outcome events or summary measures over time	9,10, 17

Main results	16	(*a*) Give unadjusted estimates and, if applicable, confounder-adjusted estimates and their precision (eg, 95% confidence interval). Make clear which confounders were adjusted for and why they were included	9,10, 19
		(*b*) Report category boundaries when continuous variables were categorized	
		(*c*) If relevant, consider translating estimates of relative risk into absolute risk for a meaningful time period	9,10,19
Other analyses	17	Report other analyses done—eg analyses of subgroups and interactions, and sensitivity analyses	10
**Discussion**			
Key results	18	Summarise key results with reference to study objectives	10,11
Limitations	19	Discuss limitations of the study, taking into account sources of potential bias or imprecision. Discuss both direction and magnitude of any potential bias	11,12
Interpretation	20	Give a cautious overall interpretation of results considering objectives, limitations, multiplicity of analyses, results from similar studies, and other relevant evidence	12
Generalisability	21	Discuss the generalisability (external validity) of the study results	11,12
**Other information**			
Funding	22	Give the source of funding and the role of the funders for the present study and, if applicable, for the original study on which the present article is based	12

Supplementary Figure 1

Non-standardized overall survival probability with time from baseline (top) and age (bottom) as time scale. The survival probability was estimated via Kaplan-Meier. Individuals at risk, censored and dead (i.e. events) are reported at the bottom of each panel. Censored individuals are those lost during follow-up when the time scale is the time from baseline, while are those lost during follow-up and censored at the end of follow-up when the time scale is age. When the time scale is age, left truncation is present until age 20.

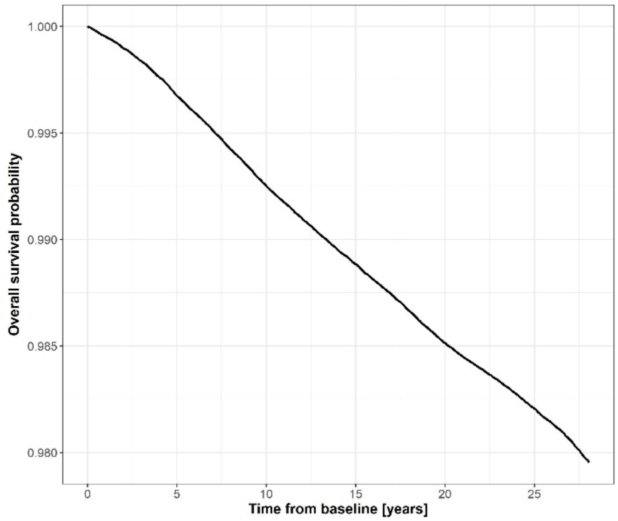

At Risk	556665	550950	428158	425301	418110
Censored	7698	11057	132257	133513	139384
Events	1828	4184	5776	7377	8697

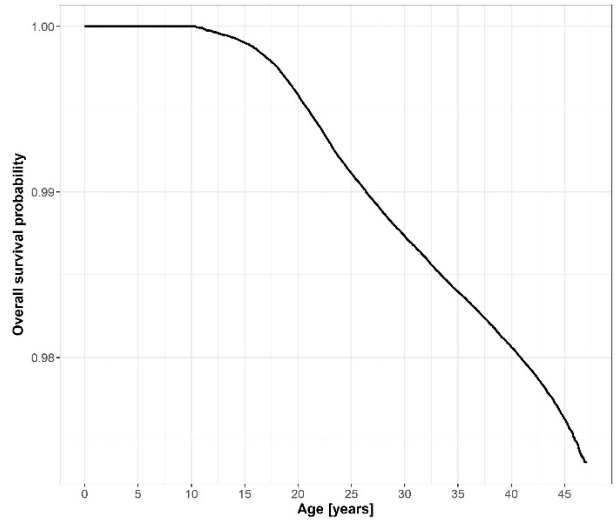

At Risk	315289	557079	475516	428117	423509	326978	102054
Censored	2365	7447	86511	132189	135349	230606	454518
Events	182	1705	4164	5885	7333	8702	9619

Supplementary Figure 2

Distribution of household crowding estimated as the ratio between the number of persons living in the household and the number of available rooms excluding kitchen and bathrooms.

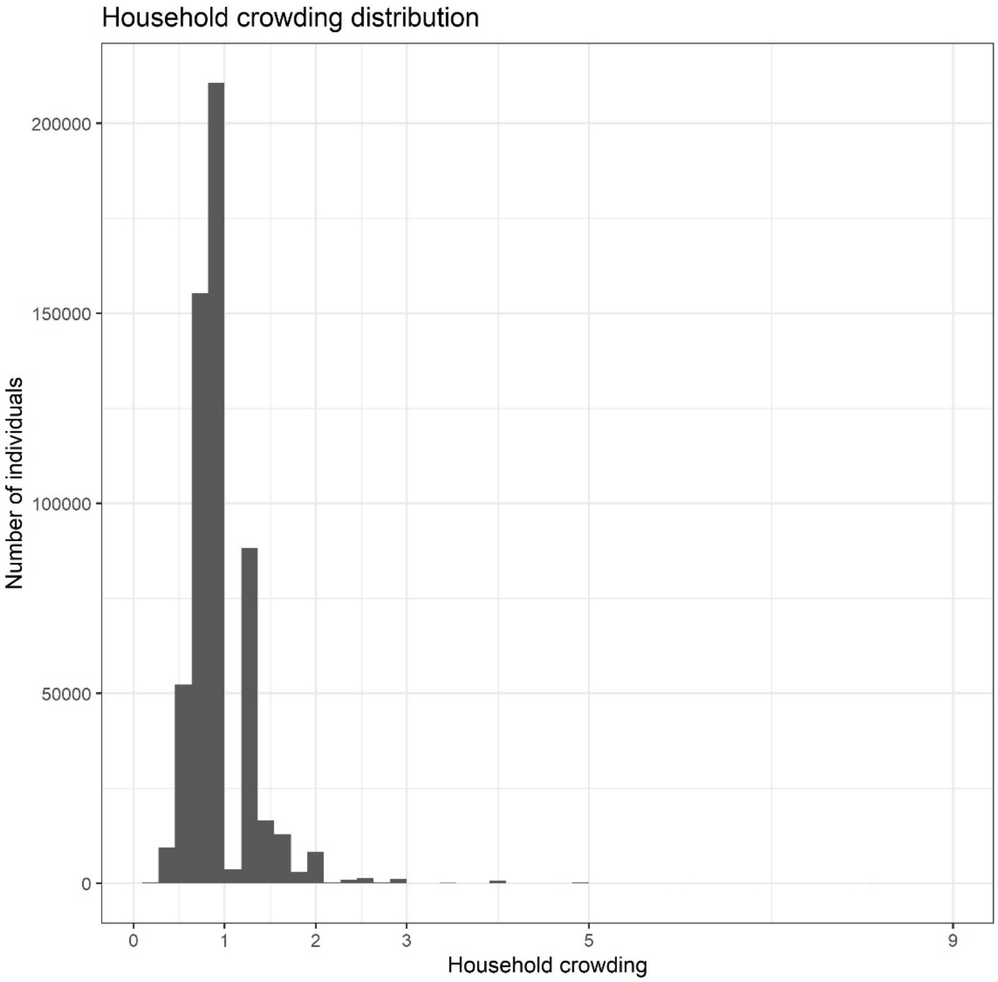

Supplementary Figure 3

Detailed causal model, where household crowding is the exposure and the outcome child premature mortality related to all causes, self-harm/substance use, and cardiometabolic disease the outcomes. Measured confounders (shown in bold) include living in a 1- or 2-parent household, parental occupation (low/high position), permit status (Swiss versus non-Swiss), and urban/non-urban area of residence. Unmeasured confounders are parental wealth, income, and child disabilities. For simplicity, censoring due to loss at follow-up is not shown.

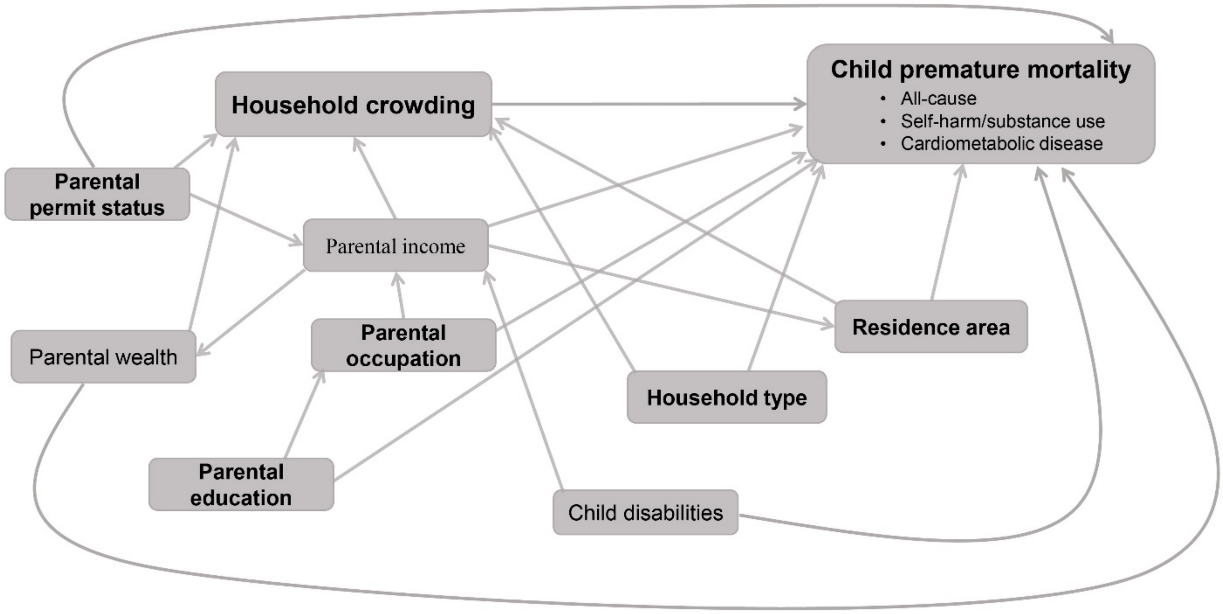

RESULTS

Supplementary Table 2

Mapping of ICD-8 and ICD-10 codes to examined death categories.

**Cause-specific death categories**	**ICD-10 codes**	**ICD-8 codes**
Cardiovascular diseases	Chapter IX<br>Diseases of the circulatory system (I00–I99)	(400–404) Hypertensive disease<br>(410–414) Ischaemic heart disease<br>(420–429) Other forms of heart disease<br>(430–438) Cerebrovascular disease<br>(440–448) Diseases of arteries, arterioles and capillaries<br>(450–458) Diseases of veins and lymphatics, and other diseases of circulatory system
Substance use disorders	F10–F10.9, G31.2, G72.1, P04.3, Q86.0, R78.0, X45–X45.9, X65–X65.9, Y15–Y15.9<br>F11–F16.9, F18–F19.9, P04.4, P96.1, R78.1–R78.5	304 Drug dependence
Diabetes, urogenital, blood, and endocrine diseases	Chapter IV<br>Endocrine, nutritional and metabolic diseases (E00–E90)<br><br>Chapter III<br>Diseases of the blood and blood-forming organs and certain disorders involving the immune mechanism (D50–D89)<br><br>Chapter XIV<br>Diseases of the genitourinary system (N00–N99)	(250–258) Diseases of other endocrine glands<br>(260–269) Avitaminoses and other nutritional deficiency<br>(270–279) Other metabolic diseases<br>(280–289) Diseases of blood and blood-forming organs
Self-harm	U03, X60–84, Y87.0	(950–959)

Supplementary Table 3

Baseline characteristics of the analytic sample and individuals lost at baseline. SMD corresponds to the standardized mean difference across the analytic and lost at baseline sample.

	**Analytic sample**<br>N = 566 191	**Lost at baseline**<br>N = 101 163	
**Sex**			SMD = 0·07
Men	294 660 (52%)	49 257 (49%)	
Women	271 531 (48%)	51 906 (51%)	
**Household type**			SMD = 0·07
2 parents	507 574 (90%)	88474 (87·5%)	
1 parent	58 617 (10%)	12689 (12·5%)	
**Parental education**			SMD = 0·11
High	167 691 (30%)	35 426 (35%)	
Low	385 486 (68%)	64 556 (64%)	
Missing	13 014 (2%)	1181 (1%)	
**Parental occupational position**			SMD = 0·20
High	454 139 (80%)	87 432 (86%)	
Low	85 221 (15%)	8727 (9%)	
Missing	26 831 (5%)	5004 (5%)	
**Parental permit of residence**			SMD = 0·46
Swiss	446 568 (79%)	95 369 (94%)	
Non-Swiss	118 697 (21%)	5726 (6%)	
Missing	926 (0·2%)	68 (0·1%)	
**Residence area**			SMD = 0·18
Urban	140 982 (25%)	17 882 (18%)	
Non-Urban	425 209 (75%)	83 281 (82%)	
**Household crowding**			SMD = 0·13
No	427 965 (76%)	81 415 (80·5%)	
Moderate	108 604 (19%)	16 246 (16%)	
Severe	29 622 (5%)	3502 (3·5%)	

Supplementary Table 4

Baseline characteristics of the analytic sample without individuals lost during follow-up and with individuals lost during follow-up. Among the individuals lost during the follow-up, N= 22 314 left the country while N = 120 723 could not be linked to mortality registers after 2000 (i.e., were right censored after 10 years of follow-up). Parental education was operationalized as previously described (10). SMD corresponds to the standardized mean difference across those not lost and lost during follow-up.

	**Analytic sample for those not lost during follow-up** N = 423 154	**Analytic sample for those lost during follow-up** N = 143 037	
**Sex**			SMD = 0·06
Men	223 322 (53%)	71 338 (50%)	
Women	199 832 (47%)	71 699 (50%)	
**Household type**			SMD = 0·01
2 parents	379 797 (90%)	127 777 (89%)	
1 parent	43 357 (10%)	15 260 (11%)	
**Parental education**			SMD = 0·12
High	119 559 (29%)	48 132 (34%)	
Low	293 473 (69%)	92 013 (64%)	
Missing	10 122 (2%)	2892 (2%)	
**Parental occupational position**			SMD = 0·05
High	336 959 (80%)	117 180 (82%)	
Low	65 581 (15%)	19 640 (14%)	
Missing	20 614 (5%)	6217 (4%)	
**Parental permit of residence**			SMD = 0·02
Swiss	332 932 (79%)	113 636 (79·5%)	
Non-Swiss	89 468 (21%)	29 229 (20·4%)	
Missing	754 (0·2%)	172 (0·1%)	
**Residence area**			SMD = 0·16
Urban	112 326 (27%)	28 656 (20%)	
Non-Urban	310 828 (73%)	114 381 (80%)	
**Household crowding**			SMD = 0·06
No	317 226 (75%)	110 739 (77%)	
Moderate	83 153 (20%)	25 451 (18%)	
Severe	22 775 (5%)	6847 (5%)	

Supplementary Table 5

Number of decedents in the population by cause of death, with and without garbage code reallocation. Numbers are counts (percentage). Level 1 and 2 garbage codes were reassigned using a data-driven prediction regression model.

	**Before garbage codes reallocation**	**After garbage codes reallocation**
**All causes**	10 500	10 500
**Self-harm/substance use**	2952 (28%)	3462 (33%)
**Cardiometabolic diseases**	649 (6%)	767 (7%)
**Other causes**	5395 (51%)	6271 (60%)
**Level 1+2 garbage codes**	1504 (14%)	0

Supplementary Table 6

Effect modification by parental occupational position, permit status, household residence area, and household type. Risk in the reference category (no crowding), risk differences per 100 000 persons by deaths related to all causes (95% confidence intervals). Risk between age 10 and 45 was standardized by all measured confounding factors except for the examined effect modifier. Risk differences were estimated in each stratum of the examined potential effect modifier. Effect modification Δ is on the additive scale. Delta is the difference of effect across strata of the potential effect modifier: Low vs High for parental occupational position, Non-Swiss vs Swiss for parental permit status, Urban vs Non-Urban for household residence area, 1-Parent vs 2-Parent for household type.

Effect modifier / Effect	**Moderate vs No crowding**	**Severe vs No crowding**
**Parental occupational position**	Low: 106 (-207 to 408)<br>High: 98 (-91 to 278)<br>Δ = 8 (-378 to 377)	Low: 494 (-9 to 1014)<br>High: 205 (-123 to 616)<br>Δ = 289 (-358 to 919)
**Parental permit status**	Non-Swiss: -116 (-333 to 125)<br>Swiss: 161 (-36 to 340)<br>Δ = -277 (-576 to 36)	Non-Swiss: 262 (-75 to 609)<br>Swiss: 273 (-85 to 711)<br>Δ = -11 (-586 to 492)
**Household residence area**	Non-Urban: 127 (-64 to 303)<br>Urban: 18 (-284 to 304)<br>Δ = -108 (-449 to 202)	Non-Urban: 159 (-163 to 548)<br>Urban: 521 (-92 to 1215)<br>Δ = 362 (-328 to 1089)
**Household type**	1-Parent: 635 (-347 to 1641)<br>2-Parent: 35 (-103 to 183)<br>Δ = 600 (-381 to 1569)	1-Parent: 1008 (-401 to 2483)<br>2-Parent: 165 (-114 to 539)<br>Δ = 842 (-666 to 2381)

Supplementary Table 7

Eurostat-based operationalization of household crowding. Risk in the reference category (no crowding), risk differences per 100 000 persons (RD) and risk ratios (RR) by deaths related to all causes (95% confidence intervals). Risk between age 10 and 45 is standardized by parental occupational position, permit status, household type, and area of residence. Eurostat-based overcrowded household corresponded to nearly 4% of the sample.

	**All causes**
**No crowding**	2351<br>(2295 to 2400)
**Yes vs. no crowding**	RD: 310 (-1 to 696)<br>RR: 1·13 (1 to 1·29)

Supplementary Table 8

Incremental truncation of inverse probability weights. Risk in the reference category (no crowding), risk differences per 100 000 persons (RD) and risk ratios (RR) by deaths related to all causes (95% confidence intervals). Risk between age 10 and 45 is standardized by parental occupational position, permit status, household type, and area of residence.

	**No truncation (main analysis)**	**Truncation percentiles 1, 99**	**Truncation percentiles 5, 95**
**No crowding**	2359<br>(2296 to 2415)	2359<br>(2295 to 2416)	2359<br>(2296 to 2416)
**Moderate vs. no crowding**	RD: 99 (-63 to 256)<br>RR: 1·04 (0·97 to 1·11)	RD: 39 (-110 to 187)<br>RR: 1·02 (0·95 to 1·08)	RD: 25 (-123 to 173)<br>RR: 1·01 (0·95 to 1·07)
**Severe vs. no crowding**	RD: 258 (-37 to 607)<br>RR: 1·11 (0·99 to 1·26)	RD: 172 (-103 to 502)<br>RR: 1·07 (0·96 to 1·21)	RD: 138 (-120 to 449)<br>RR: 1·06 (0·95 to 1·19)

Supplementary Table 9

IPWs considering individuals loss at baseline and during the follow-up. Risk in the reference category (no crowding), risk differences per 100 000 persons (RD) and risk ratios (RR) by deaths related to all causes, cardiometabolic diseases and suicide/substance use (95% confidence intervals). Risk between age 10 and 45 is standardized by parental occupational position, permit status, household type, and area of residence.

	All causes	Cardiometabolic	Suicide/Substance Use
**No crowding**	2371<br>(2305 to 2436)	209<br>(192 to 229)	766<br>(734 to 795)
**Moderate<br>vs.<br>no crowding**	RD: 98 (-74 to 270)<br>RR: 1·04 (0·97 to 1·11)	RD: -30 (-71 to 12)<br>RR: 0·86 (0·65 to 1·06)	RD: -3 (-88 to 87)<br>RR: 1·0 (0·89 to 1·12)
**Severe<br>vs.<br>no crowding**	RD: 248 (-58 to 601)<br>RR: 1·10 (0·98 to 1·25)	RD: -6 (-98 to 103)<br>RR: 0·97 (0·55 to 1·51)	RD: 13 (-159 to 202)<br>RR: 1·02 (0·79 to 1·27)

SUPPLEMENTARY REFERENCES

1. Iburg KM, Mikkelsen L, Adair T, Lopez AD. Are cause of death data fit for purpose? evidence from 20 countries at different levels of socio-economic development. PLOS ONE. 2020;15(8):e0237539.
2. Johnson SC, Cunningham M, Dippenaar IN, Sharara F, Wool EE, Agesa KM, et al. Public health utility of cause of death data: applying empirical algorithms to improve data quality. BMC Medical Informatics and Decision Making. 2021;21(1):175.
3. Rehkopf DH, Glymour MM, Osypuk TL. The Consistency Assumption for Causal Inference in Social Epidemiology: When a Rose is Not a Rose. Curr Epidemiol Rep. 2016;3(1):63-71.
4. VanderWeele TJ. Ignorability and stability assumptions in neighborhood effects research. Statistics in Medicine. 2008;27(11):1934-43.
5. Clarke P. When can group level clustering be ignored? Multilevel models versus single-level models with sparse data. Journal of Epidemiology and Community Health. 2008;62(8):752.
6. Schmidlin K, Clough-Gorr KM, Spoerri A, Egger M, Zwahlen M, for the Swiss National C. Impact of unlinked deaths and coding changes on mortality trends in the Swiss National Cohort. BMC Medical Informatics and Decision Making. 2013;13(1):1.
7. Hainmueller J. Entropy Balancing for Causal Effects: A Multivariate Reweighting Method to Produce Balanced Samples in Observational Studies. Political Analysis. 2012;20(1):25-46.
8. Greifer N. WeightIt: weighting for covariate balance in observational studies. 2022. p. https://ngreifer.github.io/WeightIt/, https://github.com/ngreifer/WeightIt.

9. Cole SR, Hernán MA. Constructing Inverse Probability Weights for Marginal Structural Models. American Journal of Epidemiology. 2008;168(6):656-64.
10. Anker D, Cullati S, Rod NH, Chiolero A, Carmeli C. Intergenerational educational trajectories and premature mortality from chronic diseases: A registry population-based study. SSM - Population Health. 2022;20:101282.
